# Unveiling the traits of HER2-low breast cancer: a comparative analysis of IHC1+ vs IHC2+/ISH-negative subgroups – insights from a 3-year cohort study

**DOI:** 10.3389/fonc.2025.1675075

**Published:** 2025-11-07

**Authors:** Jorge Correia, Catarina Pulido, Joana Albuquerque, Gil Prazeres, Inês Margarido, Mariana Câmara, Rita Neto, Gonçalo Fernandes, João Godinho, Mónica Nave, Francisco Mascarenhas, Isabel Estudante, Paulina Lopes, Ana Catarino, José Luís Passos-Coelho

**Affiliations:** 1 Department of Medical Oncology, Hospital da Luz Lisboa, Lisbon, Portugal; 2 Universidade Católica Portuguesa, Faculdade de Medicina, Lisbon, Portugal; 3 Department of Radiation Oncology, Hospital da Luz Lisboa, Lisbon, Portugal; 4 Department of Radiology, Hospital da Luz Lisboa, Lisbon, Portugal; 5 Department of Breast Surgery, Hospital da Luz Lisboa, Lisbon, Portugal; 6 Department of Pathology, Hospital da Luz Lisboa, Lisbon, Portugal

**Keywords:** IHC, ISH, PCR, HER2-low breast cancer, prognostic biomarkers, antibody-drug conjugate (ADC), real-world cohort study

## Abstract

**Background:**

Half of all breast cancer (BC) cases fall into the HER2-low category, defined as immunohistochemistry (IHC) 1+ or IHC 2+ *in situ* hybridization negative (ISH-). Two-thirds of these cases are IHC1+, while one-third is IHC2+/ISH-. New anti-HER2 antibody-drug conjugates (ADCs) have emerged as treatment options for metastatic or unresectable HER2-low BC patients. However, the heterogeneity between IHC1+ and IHC2+/ISH- subgroups and the clinical implications of varying HER2-low expression remain unclear.

**Objectives:**

This study aimed to compare demographic and clinicopathological differences between IHC1+ and IHC2+/ISH- subgroups and evaluate their response to neoadjuvant chemotherapy (NACT) in a cohort of patients with HER2-low BC.

**Methods:**

All consecutive patients diagnosed with HER2-low invasive BC between 2018 and 2020 at our institution were included in this retrospective cohort study. Clinicopathological characteristics were compared between IHC1+ and IHC2+/ISH- subgroups. Pathologic complete response (pCR) rates were assessed in patients undergoing NACT, and a multivariable logistic regression model was used to identify factors associated with pCR.

**Results:**

A total of 222 patients were included, evenly divided between IHC1+ (n=105, 47%) and IHC2+/ISH- (n=117, 53%) tumors, with no significant differences in baseline characteristics. Both subgroups predominantly comprised female patients (99% IHC1+ vs. 98% IHC2+/ISH-), postmenopausal (55% vs. 58%), with early-stage BC (94% vs. 98%) and estrogen receptor (ER)-positive tumors (90% vs. 90%). Around two-thirds had grade 2 tumors (63% vs. 64%), and the median Ki-67 index was 20% in both subgroups. Most BC were classified as luminal B-like (56% vs. 58%), followed by luminal A-like (35% vs. 34%), and TNBC (9% vs. 8%). Among the 43 patients with HER2-low BC who received NACT, 36% of IHC1+ patients achieved pCR, compared to only 5% in the IHC2+/ISH- subgroup (p = 0.021). Multivariable analysis revealed that IHC2+/ISH- status (vs. IHC1+) was significantly associated with lower odds of pCR (OR=0.07, 95% CI: 0.00–0.51, p = 0.025), while higher baseline Ki-67 and ER-negative status showed non-significant trends toward higher pCR rates after adjustment for other variables.

**Conclusion:**

Despite similar clinicopathological features, IHC2+/ISH- status was independently associated with lower pCR rates compared to IHC1+. These findings suggest that HER2-low subgroups may influence response to NACT and should be considered in multivariable prediction models, potentially informing stratified treatment approaches in the era of anti-HER2 ADCs.

## Introduction

Breast cancer (BC) is the second most common cancer worldwide and remains the leading malignancy among women. In 2022, it accounted for approximately 2.3 million new cases and 700,000 deaths worldwide, making it the primary cause of cancer-related mortality in females (1).

Breast tumors are routinely classified by estrogen receptor (ER), progesterone receptor (PR), and human epidermal growth factor receptor 2 (HER2) status, with ER/PR defining hormone receptor–positive disease and HER2 defining HER2−positive disease; within the HER2−negative category, HER2−low has recently emerged as a clinically relevant subset.

The HER2 is a transmembrane tyrosine kinase receptor that significantly affects cell growth and proliferation. Beyond their role in molecular subtyping, HER family receptors are clinically important cell−surface markers that correlate with tumor biology and radiologic phenotypes, supporting their integration into diagnostic and therapeutic decision−making (51). Overexpression driven by gene amplification correlates with a more aggressive tumor phenotype, increased recurrence risk, and historically poorer outcomes (2–7) — a reality mitigated by the advent of HER2-targeted therapies, which have transformed early-stage HER2-positive BC into a highly curable disease and significantly improved survival in the metastatic context (2).

Formerly, HER2 status was classified in a binary fashion: ‘positive’ for overexpression (immunohistochemistry [IHC] 3+) or amplification (positive *in situ* hybridization [ISH]), and ‘negative’ when these alterations were absent (IHC scores of 0, 1+, or 2+/ISH-negative [2+/ISH-]) (3–5). Based on this paradigm, about 15% of BCs were HER2-positive, while the remaining 85% were classified as HER2-negative, many of which still harbor detectable HER2 protein by IHC (8).

HER2-negative tumors have been classified and treated based on hormone receptor (HR) IHC staining results, dividing it into two primary clinical subtypes: HR-positive (ER and/or PR-positive, HER2-negative) and triple-negative (ER, PR, and HER2-negative) (9).

More recently, distinct subsets within the HER2-negative category have been proposed: ‘HER2-low’ for tumors with IHC scores of 1+ or 2+/ISH-, and ‘HER2-null’ for those with no detectable staining (IHC score of 0) (2). Additionally, the term ‘HER2-ultralow’ has been suggested for tumors with faint staining below 1+, which are currently included in the HER2-null category (10).

Despite some limitations of available methods, IHC and ISH remain the gold standard for identifying HER2-low BC and guiding treatment decisions. Existing evidence does not provide sufficient basis to attribute meaningful predictive value to variations in HER2 IHC levels, and clinical guidelines currently recommend treating both IHC 1+ and IHC 2+/ISH- subgroups as a single entity.

Given the significant prevalence of HER2-low BC, a deeper understanding of the heterogeneity within this subset and its impact on disease progression is crucial for refining breast cancer management strategies. Most previous trials involving HER2-negative BC did not provide detailed information on HER2-low status, making retrospective cohort studies particularly valuable in this context. Moreover, comparative studies between HER2-low 1+ and 2+ subgroups are lacking.

To address these concerns, we conducted a retrospective analysis of patients diagnosed with HER2-low invasive BC at our center. Our primary objective was to explore demographic and clinicopathological differences between two HER2-low subgroups (IHC1+ vs. IHC2+/ISH-). Our secondary objective was to evaluate the differential response to neoadjuvant chemotherapy (NACT) among patients treated with this strategy, focusing on the association between HER2-low status and pathologic complete response (pCR). Eventually, this study aims to contribute to ongoing efforts in improving risk stratification, prognostication, and development of tailored treatment protocols for patients with HER2-low BC.

## Materials and methods

### Study design

This investigator−initiated retrospective cohort study was conducted at Hospital da Luz Lisboa (Lisbon, Portugal), a private, university−affiliated tertiary hospital with integrated oncology services and accredited fellowship training, with care aligned to national and European breast−cancer guidelines. Eligible patients had histologically confirmed HER2−low invasive breast cancer (IHC 1+ or IHC 2+/ISH−) diagnosed between January 2018 and December 2020. The primary endpoint was the comparison of baseline demographic and clinicopathological characteristics between the IHC1+ and IHC2+/ISH- subgroups, including sex, age at diagnosis, menopausal status, baseline performance status, tumor histopathological subtype, histological grade, Ki-67 expression, hormone receptor status, and clinical and pathological stages. The secondary endpoint was the comparison of pCR rates between the IHC1+ and IHC2+/ISH- subgroups who underwent NACT. Additionally, an exploratory objective was to examine variations in nodal status following NACT, from baseline to surgery after NACT across HER2−low subgroups.

### Patients

All patients diagnosed with HER2-low BC at our institution between January 2018 and December 2020 were screened. Inclusion criteria were: (1) age ≥ 18 years and (2) histologically confirmed HER2-low invasive BC (IHC1+ or IHC2+/ISH-). Patients were eligible irrespective of neoadjuvant treatment status. The exclusion criteria were: (1) pregnancy, (2) psychiatric disorders or impaired cognitive function, and (3) incomplete demographic, clinical, treatment, or pathological data in electronic medical records (EMR).

Completeness of data in the EMR was available for all patients. No exclusions occurred for pregnancy or psychiatric/cognitive conditions, which were pre−specified per the local IRB policy. All 222 screened patients met eligibility and were included in the analysis cohort.

### Data collection

Data were collected from the hospital EMR and included information up until the last documented visit.

Demographic and clinical variables included sex, age at diagnosis, date of diagnosis, menopausal status, baseline Eastern Cooperative Oncology Group Performance Status (ECOG PS), and tumor clinical stage at diagnosis as per the American Joint Committee on Cancer (AJCC) 8th edition of the tumor, node, metastasis (TNM) staging system.

Treatment and pathological variables comprised tumor histopathological subtype (non-special-type [NST], infiltrating lobular carcinoma [ILC], or other), World Health Organization (WHO) histological grade (1, 2, or 3), IHC Ki-67 expression, HR status (including ER and PR status), pathological stage at surgery according to the AJCC 8th edition TNM staging system, type of chemotherapy received and pCR status at the time of surgery for those who underwent NACT.

### Pathology

HER2, ER, PR status, and Ki-67 index were obtained from the original pathology reports and evaluated according to the 2018 ASCO/CAP guidelines (3). Surgical samples were preferred over biopsy specimens, except in cases undergoing NACT, where baseline core biopsies of the primary tumor were used instead. HER2 status was considered positive if scored as 3+ by IHC or 2+ with HER2 amplification by ISH. Conversely, HER2 was classified as negative if the score was 0 or 1+ by IHC, or 2+ without HER2 amplification by ISH. Within HER2-negative cases, further subclassification was applied: ‘HER2-null’ referred to an IHC score of 0, and ‘HER2-low’ to scores of 1+ or 2+ in the absence of HER2 gene amplification by ISH. Both ER and PR were considered positive if IHC staining was present in ≥1% of cancer cells. HR status was classified as positive (HR+) if either ER or PR tested positive, and negative if both ER and PR were negative. The clinicopathologic surrogate definitions of intrinsic breast cancer subtypes, based on IHC, followed the 2013 St. Gallen Consensus Conference guidelines (11): luminal A-like for ER positivity, high PR (≥20% of tumor cells staining positive), HER2 negativity, and a low Ki-67 index (<20%); luminal B-like HER2-negative for ER positivity, along with either low PR (<20%) or a high Ki-67 index (≥20%); triple-negative breast cancer (TNBC) for the absence of ER, PR, and HER2 expression. In patients undergoing NACT, pCR was defined as the absence of any residual invasive carcinoma in both the breast and axillary lymph nodes (ypT0/Tis ypN0), as determined by histopathological evaluation after the completion of neoadjuvant treatment.

### Ethical considerations

This investigator-initiated study received approval from our center’s Institutional Review Board/Independent Ethics Committee (IRB/IEC) and was designed according to the ethical principles of the Declaration of Helsinki and Good Clinical Practice guidelines. Clinical data were treated with pseudonymization to ensure strict confidentiality, were accessible only to the primary investigator, and were used exclusively within the scope of this research. A waiver of informed consent was requested by the investigators and granted by the IRB/IEC due to the retrospective and non-interventional nature of the study.

### Financial disclosure

Participants incurred no costs for their involvement in the study. Data analysis was conducted by the research team, with all associated costs covered by the investigators. No external funding was granted, and publication fees were supported internally to ensure comprehensive dissemination of our findings to the broader scientific community.

### Statistical analysis

Categorical variables (sex, age group, menopausal status, ECOG PS, clinical stage, histopathological subtype, grade, HER2 score, HR status and Ki-67 expression intervals) were summarized as frequencies. HR status was classified as positive (if either ER or PR were positive) versus negative (if both ER and PR were negative), and Ki-67 index as high (≥20%) versus low (<20%). Comparisons between HER2-low IHC 1+ and IHC 2+/ISH- subgroups were conducted using Pearson’s chi-square or Fisher’s exact tests as appropriate. In patients who underwent NACT, the association between HER2-low subgroups (IHC 1+ vs. IHC 2+/ISH-) and pCR was assessed using the chi-squared or Fisher’s exact test, as appropriate. Additionally, the association between Ki-67 expression, as a continuous variable, and pCR was evaluated using the Mann-Whitney U test or an independent sample t-test, depending on whether the data distribution was non-normal or normal, respectively. For the NACT-treated subset, univariable logistic regression constituted the primary analysis for pCR as a secondary endpoint, with effect estimates and two-sided P-values reported for each covariate of interest. The dependent variable was pCR occurrence, while the independent variables included age at diagnosis, sex, ECOG PS, clinical TNM stage, tumor histological grade, Ki-67 expression (categorized and continuous), hormone receptor status, and pathologic surrogates of intrinsic subtypes. Chemotherapy regimen was summarized descriptively and not included as a covariate in adjusted models to avoid over−parameterization. To contextualize potential confounding, a pre−specified multivariable model was deliberately limited to clinically grounded covariates using purposeful selection informed by an initial univariable screen at P<0.10 and clinical rationale (HER2−low subgroup IHC 1+ vs IHC 2+/ISH−, ER status, and baseline Ki−67 modeled continuously). Given the limited number of pCR events, this adjusted model was framed as exploratory and supportive of the univariable findings, and is interpreted alongside them. An exploratory objective was pre−specified to examine changes in axillary nodal status from diagnosis (cN) to surgery (ypN) after NACT across HER2−low subgroups (IHC 1+ vs IHC 2+/ISH−); transitions were visualized using a Sankey diagram, and differences were compared using Fisher’s exact test. All statistical tests were two−sided with α=0.05. Analyses were performed in R Software v4.1.1.

## Results

### Full cohort analysis of HER2-low invasive BC

From January 2018 to December 2020, a total of 222 consecutive patients diagnosed with HER2-low BC were screened, all of whom met eligibility criteria: 105 (47%) were classified with IHC1+ and 117 (53%) with IHC2+/ISH-. No patients were excluded.

There were no differences in demographic and clinicopathological features, between the IHC1+ and IHC2+/ISH- subgroups (**Table 1**).

**Table 1:** Baseline demographics and clinicopathological characteristics.

Characteristic	Overall N = 222^1^	HER2-low subgroups		P-value^2^
		IHC 1+ N = 105^1^	IHC 2+/ISH- N = 117^1^	
Sex				1
Female	219 (99%)	104 (99%)	115 (98%)	
Male	3 (1.4%)	1 (1%)	2 (2%)	
Age – years (median; range)	60 (47, 71)	60 (45, 70)	60 (47, 71)	0.501
Age at diagnosis (years)				0.515
<50	72 (32%)	37 (35%)	35 (30%)	
50-69	89 (40%)	38 (36%)	51 (44%)	
≥ 70	61 (27%)	30 (29%)	31 (26%)	
Performance Status (ECOG)				0.932
0-1	196 (88%)	92 (88%)	104 (89%)	
2-4	26 (12%)	13 (12%)	13 (11%)	
Menopausal Status				0.705
Postmenopausal	124 (57%)	57 (55%)	67 (58%)	
Premenopausal	95 (43%)	47 (45%)	48 (42%)	
cT				0.810
1	130 (59%)	62 (59%)	68 (58%)	
2	65 (29%)	29 (28%)	36 (31%)	
3-4	27 (12%)	14 (13%)	13 (11%)	
cN				1
0	172 (77%)	81 (77%)	91 (78%)	
≥ 1	50 (23%)	24 (23%)	26 (22%)	
cM				0.1534
0	214 (96%)	99 (94%)	115 (98%)	
1	8 (3.6%)	6 (5.7%)	2 (1.7%)	
Histologic type				0.308
NST	169 (76%)	84 (80%)	85 (73%)	
ILC	34 (15%)	12 (11%)	22 (19%)	
Other	19 (8.6%)	9 (8.6%)	10 (8.5%)	
Histologic grade				0.839
1	41 (18%)	21 (20%)	20 (17%)	
2	141 (64%)	66 (63%)	75 (64%)	
3	40 (18%)	18 (17%)	22 (19%)	
ER				1
Positive	200 (90%)	95 (90%)	105 (90%)	
Negative	22 (9.9%)	10 (9.5%)	12 (10%)	
PR				1
Positive	193 (87%)	91 (87%)	102 (87%)	
Negative	29 (13%)	14 (13%)	15 (13%)	
Ki-67 index (%) - (median; range)	20 (10, 40)	20 (10, 40)	20 (10, 40)	0.692
Baseline Ki-67 index (%)				0.588
< 20	92 (41%)	46 (44%)	46 (39%)	
≥ 20	130 (59%)	59 (56%)	71 (61%)	
Intrinsic subtype				0.948
Luminal A-like	77 (35%)	37 (35%)	40 (34%)	
Luminal B-like	127 (57%)	59 (56%)	68 (58%)	
TNBC	18 (8.1%)	9 (8.6%)	9 (7.7%)	
Neoadjuvant Chemotherapy (NACT)				0.693
No	179 (81%)	83 (79%)	96 (82%)	
Yes	43 (19%)	22 (21%)	21 (18%)	

ILC, Infiltrating lobular carcinoma; ECOG, Eastern Cooperative Oncology Group; ER, Estrogen receptor; IHC, Immunohistochemistry; ISH, *In situ* hybridization; M, Metastasis; N, Node; NACT, Neoadjuvant chemotherapy; NST, No-special-type; PR, Progesterone receptor; T, Tumor; TNBC, Triple-negative breast cancer.

^1^n (%); Median (Q1, Q3).

^2^Pearson’s Chi-squared test; Fisher’s exact test; Wilcoxon rank sum test.

The cohort predominantly comprised female patients (99% vs. 98%), most of whom were postmenopausal (55% vs. 58%), with the same median age of 60 years. Most patients had a favorable overall functional status, with an ECOG PS score of 0-1 (88% vs. 89%), and early-stage BC, characterized by T1 (59% vs. 58%), N0 (77% vs. 78%), and M0 (94% vs. 98%) tumors. A small proportion of patients (3.6%) had metastatic disease, with bone and liver as the most common sites, each present in 50% of these cases.

Histopathological analysis revealed that most tumors were NST (80% vs. 73%), with ILC as the second most prevalent histologic type (11% vs. 19%). The majority of tumors were ER (90% vs. 90%) and PR (87% vs. 87%) positive, two-thirds were classified as G2 (63% vs. 64%), and median Ki-67 was 20% for both IHC1+ and IHC2+/ISH- subgroups.

There was a similar breakdown of the surrogates of intrinsic subtypes, with tumors primarily classified as luminal B-like (56% vs. 58%), followed by luminal A-like (35% vs. 34%), and TNBC (9% vs. 8%).

### Focused analysis of HER2-low BC treated with NACT

Of the 222 patients, a subset of 43 (19.4%) underwent NACT prior to surgery: 22 (51%) with IHC1+ and 21 (49%) with IHC2+/ISH- tumors. Of 43 patients, 37 (86%) received a standard dose−dense doxorubicin-cyclophosphamide (AC) sequence followed by paclitaxel, with carboplatin added for TNBC as clinically indicated; distribution across HER2−low subgroups was balanced (IHC1+ n=20; IHC2+/ISH− n=17) (**Table 2**). The remaining six patients received other regimens. In IHC2+/ISH−: cyclophosphamide-methotrexate-fluorouracil (CMF) (n=2), docetaxel−cyclophosphamide (TC) (n=1), carboplatin+paclitaxel (n=1); and in IHC1+: TC (n=1), fluorouracil-epirubicin-cyclophosphamide followed by docetaxel (FEC−D/PACS−01) (n=1). None were treated with PD-1/PD-L1 inhibitors.

**Table 2:** Baseline demographics and clinicopathological characteristics - NACT-treated cohort.

Characteristic	Overall N = 43^1^	HER2-low status		P-value^2^
		IHC 1+ N = 22^1^	IHC 2+/ISH- N = 21^1^	
Sex				
Female	43 (100%)	22 (100%)	21 (100%)	
Age – years (median, range)	50 (43, 68)	45 (41, 53)	57 (47, 68)	**0.006**
Age at diagnosis (years)				0.138
<50	21 (49%)	14 (64%)	7 (33%)	
50-69	14 (33%)	5 (23%)	9 (43%)	
≥ 70	8 (19%)	3 (14%)	5 (24%)	
Performance status (ECOG)				
0-1	43 (100%)	22 (100%)	21 (100%)	
2-4	0 (0%)	0 (0%)	0 (0%)	
Menopausal Status				**0.004**
Postmenopausal	18 (42%)	4 (18%)	14 (67%)	
Premenopausal	25 (58%)	18 (82%)	7 (33%)	
cT				0.596
1	4 (9.3%)	3 (14%)	1 (4.8%)	
2	22 (51%)	11 (50%)	11 (52%)	
3-4	17 (40%)	8 (36%)	9 (43%)	
cN				0.843
0	16 (37%)	9 (41%)	7 (33%)	
≥ 1	27 (63%)	13 (59%)	14 (67%)	
cM				1
0	42 (98%)	21 (95%)	21 (100%)	
1	1 (2.3%)	1 (4.5%)	0 (0%)	
Histological type				0.610
NST	34 (79%)	17 (77%)	17 (81%)	
ILC	7 (16%)	3 (14%)	4 (19%)	
Other	2 (4.7%)	2 (9.1%)	0 (0%)	
Histological grade				0.598
1	2 (4.7%)	2 (9.1%)	0 (0%)	
2	23 (53%)	11 (50%)	12 (57%)	
3	18 (42%)	9 (41%)	9 (43%)	
ER				1
Positive	27 (63%)	14 (64%)	13 (62%)	
Negative	16 (37%)	8 (36%)	8 (38%)	
PR				0.902
Positive	26 (60%)	14 (64%)	12 (57%)	
Negative	17 (40%)	8 (36%)	9 (43%)	
Ki67 index – % (median, range)	60 (25, 80)	60 (20, 80)	40 (25, 75)	0.836
Ki67 index (%)				0.664
≥ 20	37 (86%)	18 (82%)	19 (90%)	
< 20	6 (14%)	4 (18%)	2 (9.5%)	
Intrinsic subtypes				0.992
Luminal A-like	4 (9.3%)	2 (9.1%)	2 (9.5%)	
Luminal B-like	25 (58%)	13 (59%)	12 (57%)	
TNBC	14 (33%)	7 (32%)	7 (33%)	
NACT protocol				0.412
AC-based	37 (86%)	20 (91%)	17 (81%)	
Other	6 (14%)	2 (9.1%)	4 (19%)	
pCR				**0.021**
Yes	9 (21%)	8 (36%)	1 (4.8%)	
No	34 (79%)	14 (64%)	20 (95%)	

ILC, Infiltrating lobular carcinoma; ECOG, Eastern Cooperative Oncology Group; ER, Estrogen receptor; IHC, Immunohistochemistry; ISH, *In situ* hybridization; M, Metastasis; N, Node; NACT, Neoadjuvant chemotherapy; NST, No-special-type; pCR, Pathologic complete response; PR, Progesterone receptor; T, Tumor; TNBC, Triple-negative breast cancer.

^1^n (%); Median (Q1, Q3).

^2^Wilcoxon rank sum test; Pearson’s Chi-squared test. Bold values indicate statistical significance (p ≤ 0.05).

All patients were female with an ECOG PS score of 0-1. The median age was lower in the IHC1+ subgroup compared to the IHC2+/ISH- subgroup (45 vs. 57 years, p = 0.006), and fewer were postmenopausal (18% vs. 67%, p = 0.004).

No significant differences were observed in the clinical stage distribution, with both IHC1+ and IHC2+/ISH- subgroups predominantly presenting with higher stage disease, primarily as T2 (50% vs. 52%) and N1-2 (59% vs. 67%).

Histopathological analysis showed that the most prevalent subtype in both subgroups was NST (77% vs. 81%), followed by ILC (14% vs. 19%). Approximately two-thirds of tumors were ER-positive (64% vs. 62%) and PR-positive (64% vs. 57%). Nearly all tumors were classified as grade ≥ 2, with almost half falling into the G3 category (41% vs. 43%).

Despite some variability, baseline Ki-67 index levels were generally elevated on both IHC1+ and IHC2+/ISH-, with no statistically significant differences detected between subgroups.

Furthermore, a similar distribution of clinicopathologic surrogates of intrinsic subtypes was observed between the IHC1+ and IHC2+/ISH- subgroups: more than half of tumors were classified as luminal B-like (59% vs. 57%), about one-third as TNBC (32% vs. 33%), and a smaller fraction as luminal A-like (9% vs. 10%).

### Assessment of pCR in NACT-treated cohort

**Figure 1:**
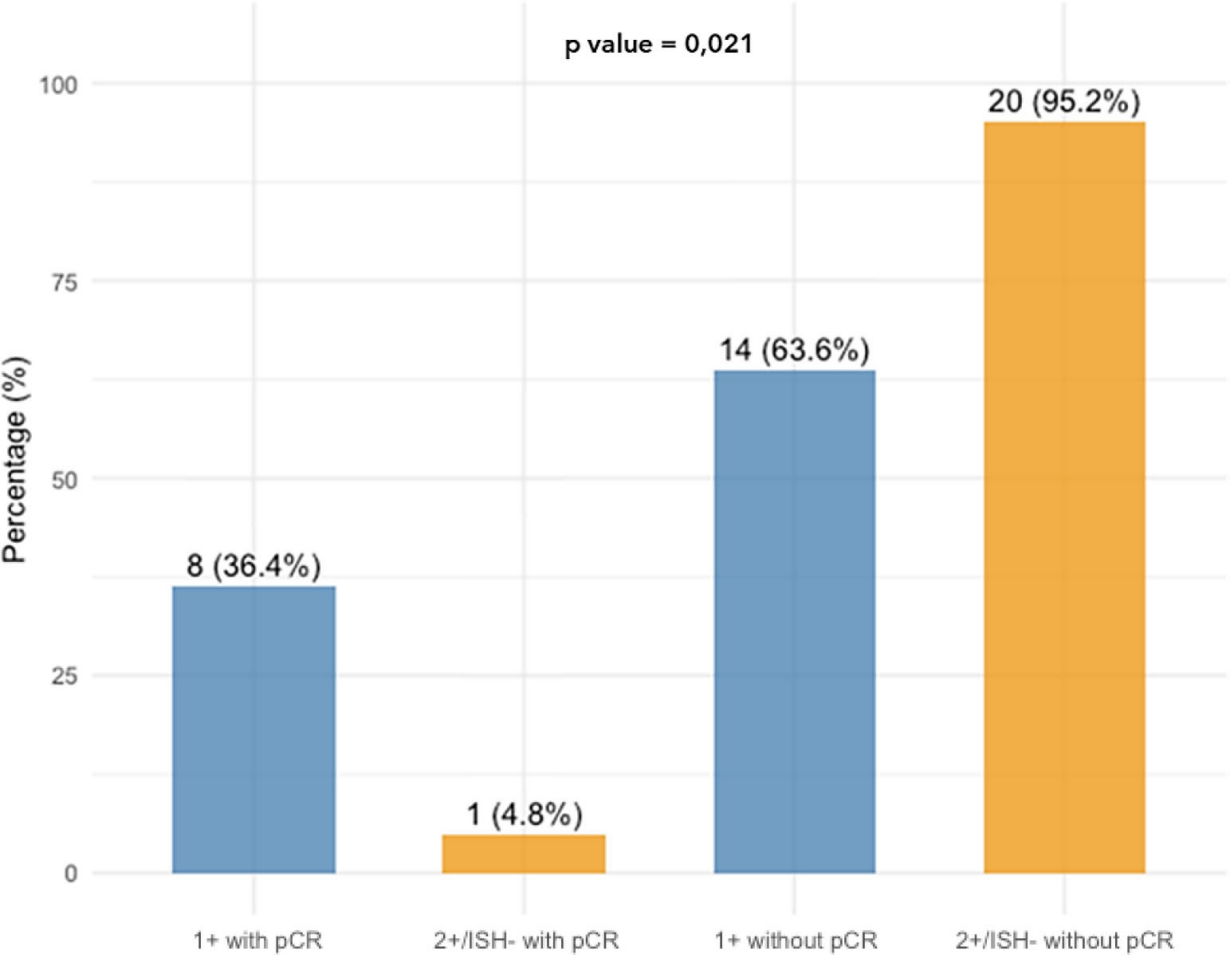
Bar chart showing the distribution of pCR rates based on HER2-low status. Among IHC 1+ patients, 36.4% (8 out of 22) achieved pCR. In contrast, among IHC 2+/ISH- patients, only 4.8% (1 out of 21) achieved pCR. This illustrates a statistically significant difference in pCR rates between the two subgroups (p =0.021). IHC, Immunohistochemistry; ISH, *In situ* hybridization; pCR, Pathologic complete response.

The pCR rate was 36% in patients with IHC1+ tumors (8 out of 22) compared to only 5% (1 out of 21) of IHC2+/ISH- patients (p = 0.021; **Table 2**, **Figure 1**).

**Figure 2:**
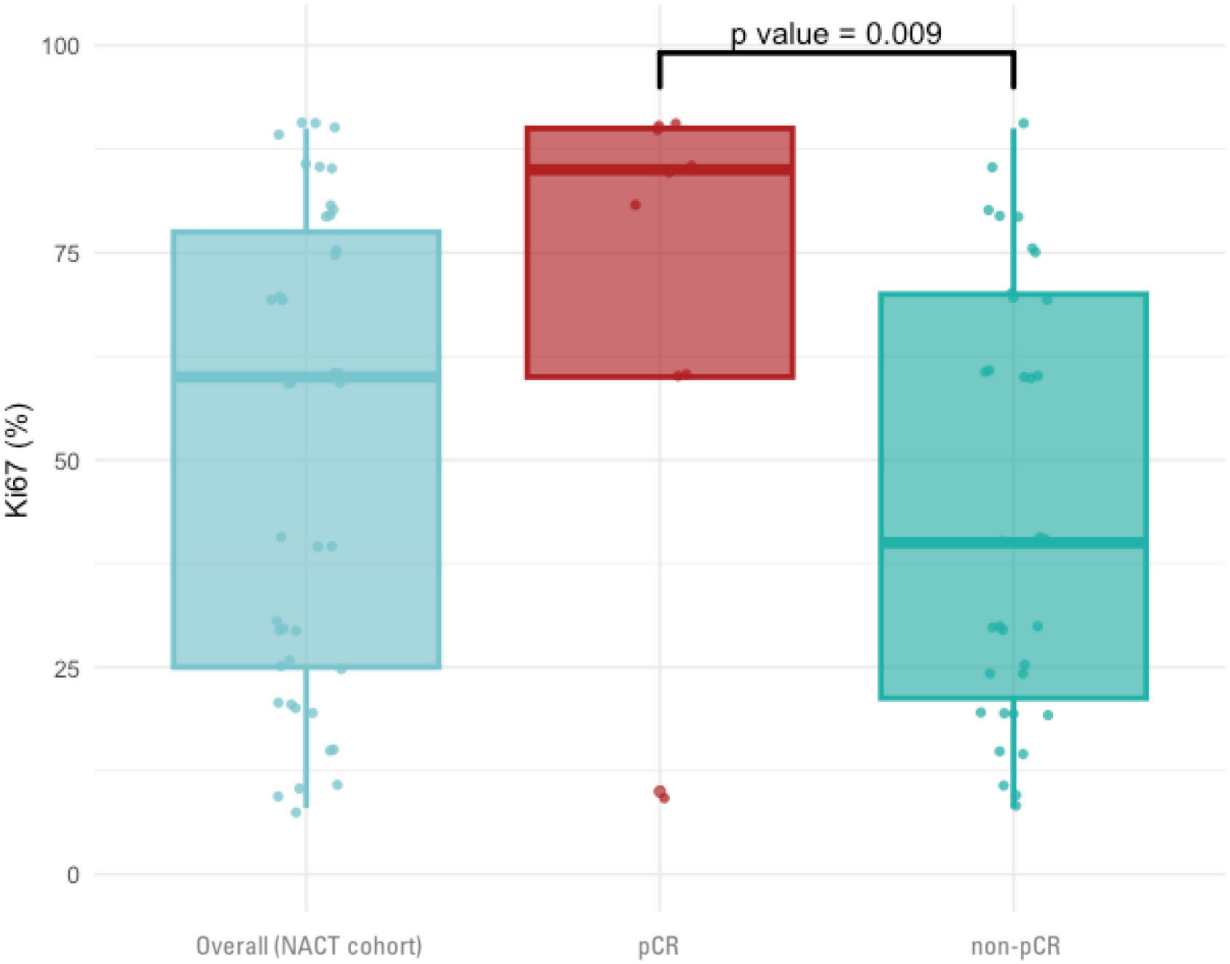
Box plots illustrating the distribution and median Ki-67 levels measured in the diagnostic biopsy in three groups: the overall cohort of patients treated with NACT, those who achieved a pCR, and those who did not achieve pCR (non-pCR). A statistically significant difference in Ki-67 levels between the pCR and non-pCR groups is noted NACT, Neoadjuvant chemotherapy; pCR, Pathologic complete response.

The median baseline Ki-67 level was higher among individuals who achieved pCR after NACT compared to those who did not (median Ki-67 of 85% vs. 40%; p = 0.009; **Figure 2**).

The relationship between clinicopathological variables and pCR was examined using a logistic regression model. In univariable analysis, IHC 2+/ISH− (vs IHC 1+) and ER−positive (vs ER−negative) status were associated with lower odds of pCR (OR 0.09, 95% CI 0.00–0.55, P=0.029; and OR 0.21, 95% CI 0.04–0.95, P=0.050, respectively), whereas higher baseline Ki−67 (as a continuous variable) was associated with higher odds of pCR (OR 1.05 per 1% increase, 95% CI 1.01–1.09, P=0.021). In the pre−specified multivariable model — limited to HER2−low subgroup, ER status, and baseline Ki−67 per the purposeful−selection plan — only the association for IHC 2+/ISH− remained statistically significant (OR 0.07, 95% CI 0.00–0.51, P=0.025), while ER status and Ki−67 showed non−significant trends after adjustment (**Table 3**).

**Table 3:** Univariable and multivariable logistic regression model for pCR outcome.

Characteristic	Univariable			Multivariable		
	OR	95% CI	P-value	OR	95% CI	P-value
ER Status						
Negative	1.00	Ref.		1.00	Ref.	
Positive	0.21	0.04, 0.95	**0.050**	0.33	0.04, 2.35	0.276
HER2-low subgroups						
IHC 1+	1.00	Ref.		1.00	Ref.	
IHC 2+/ISH-	0.09	0.00, 0.55	**0.029**	0.07	0.00, 0.51	**0.025**
Ki67 index (continuous)	1.05	1.01, 1.09	**0.021**	1.04	1.00, 1.10	0.085
Ki67 index (categorical intervals)						
Ki67<20%	1.00	Ref.				
Ki67 ≥ 20%	1.38	0.18, 28.4	0.783			
Age	0.97	0.90, 1.03	0.293			
ECOG PS	0.41	0.02, 2.74	0.427			
cT						
1	1.00	Ref.				
2	0.29	0.03, 2.95	0.275			
3-4	0.13	0.01, 1.59	0.107			
cN						
0	1.00	Ref.				
>=1	0.38	0.08, 1.71	0.209			
PR status						
Negative	1.00	Ref.				
Positive	0.44	0.09, 1.94	0.276			
Histologic grade						
1	1.00	Ref.				
2/3	0.24	0.01, 6.57	0.334			
Menopausal status						
Postmenopausal	1.00	Ref.				
Premenopausal	1.58	0.35, 8.47	0.562			
Intrinsic subtypes						
Luminal A-like	1.00	Ref.				
Luminal B-like	0.41	0.04, 9.73	0.495			
TNBC	1.67	0.16, 38.8	0.690			

CI, Confidence interval; ILC, Infiltrating lobular carcinoma; ECOG, Eastern Cooperative Oncology Group; ER, Estrogen receptor; IHC, Immunohistochemistry; ISH, *In situ* hybridization; N, Node; NST, Non-special-type; OR, Odds ratio; pCR, Pathologic complete response; PR, Progesterone receptor; T, Tumor; TNBC, Triple-negative breast cancer.Bold indicates statistical significance (95% CI excluding 1).

**Figure 3:**
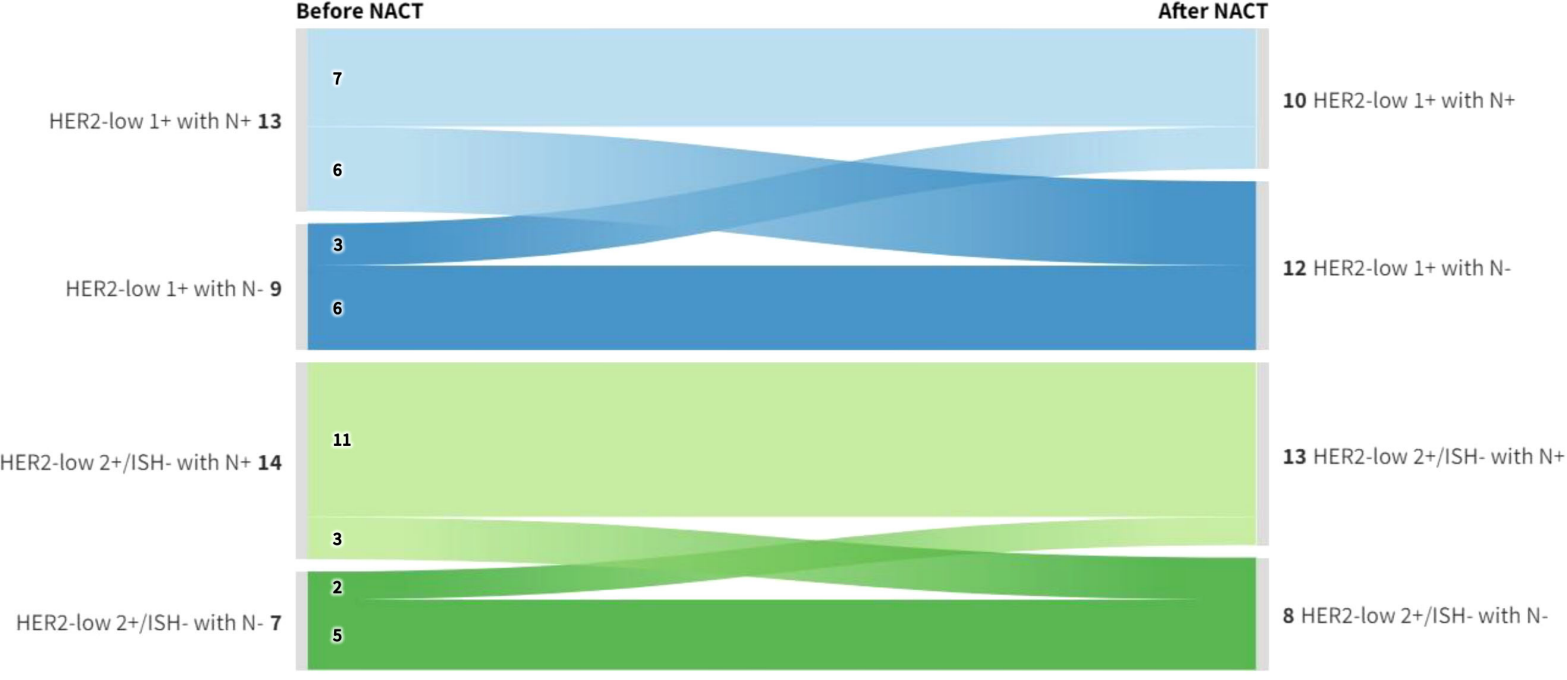
Sankey diagram depicting the variation in nodal status before and after NACT across HER2-low subgroups. The diagram illustrates the distribution of nodal status (positive [N+] and negative [N-]) in both IHC 1+ and IHC 2+/ISH- patients before and after NACT. Despite the observed shifts, no statistically significant differences were found between the HER2-low subgroups concerning changes in nodal status following NACT (p = 0.384). IHC, Immunohistochemistry; ISH, *In situ* hybridization; NACT, Neoadjuvant chemotherapy.

An exploratory analysis was conducted to assess variations in nodal status (**Figure 3**). The Sankey diagram shows no statistically significant differences regarding changes in nodal status between the HER2-low subgroups after NACT (p = 0.384). Among patients presenting with cN+ disease, 6/13 IHC 1+ cases converted to ypN0 versus 3/14 in the IHC 2+/ISH− subgroup.

## Discussion

Previous studies suggest that approximately half of all BC tumors fall into the HER2-low category (8), with two-thirds scoring as IHC 1+ and one-third as IHC2+/ISH-, irrespective of HR status (12). In our cohort, there was almost an even breakdown between IHC1+ and IHC2+/ISH- subgroups (47% vs. 53%) among the 222 patients included in this retrospective analysis.

Inconsistencies in the literature regarding varying HER2 levels may stem from inaccuracies in IHC, which might confound subsequent outcome analyses (13). Originally designed to identify trastuzumab eligibility, HER2 IHC testing was not intended to discriminate between null and low HER2 levels, as this distinction had no treatment implications until recently. This may explain the suboptimal agreement among pathologists in distinguishing between IHC scores of 0 and 1+. Some reports show only a 15% concordance rate between pathologists when evaluating cases initially classified as HER2-null, with most being reclassified as IHC 1+ upon reevaluation (14). Contributing factors such as formalin fixation artifacts or insufficient sensitivity of IHC semiquantitative assays may yield false negatives (15, 16).

HER2 expression may also vary within a tumor, across metastatic sites, and between primary and recurrent tumors. Studies indicate that up to 15% of cases can shift between HER2 null and HER2-low upon recurrence (17). The influence of intratumor heterogeneity—both spatial and temporal—as well as microenvironment factors and therapeutic effects on HER2 IHC evaluation, warrant continual reassessment of HER2 status throughout the course of the disease to ensure optimal treatment sequencing (8, 15, 18).

Beyond classical immunohistochemistry, *in situ* hybridization techniques – including chromogenic ISH (CISH) – could provide complementary confirmation of HER2 gene status, particularly in IHC 2+ cases, and strengthen the analytic validity and reproducibility of HER2 classification in routine practice (e.g., alongside FISH−based approaches) (53). To overcome existing limitations, novel assays (such as quantitative IHC platforms, immunofluorescence−based automated quantitative analysis, and AI−assisted HER2 scoring) are under development to enhance reliability and reproducibility (15, 19, 20). In the meantime, adherence to ASCO/CAP guidelines is essential for accurate HER2 scoring (3).

Our primary findings revealed no significant differences in demographic or clinicopathological characteristics when comparing the IHC1+ and IHC2+/ISH- subgroups. This aligns with previous findings suggesting that HER2-low BC does not qualify as a distinct entity but is instead included within the HER2-negative spectrum. Moreover, when evaluating survival outcomes between HER2-low and HER2-null BC, most retrospective data reveal only minimal differences after adjusting for HR expression (2, 12, 16, 21–25).

A key criterion for defining a new entity is the presence of distinct genomic alterations. So far, no significant molecular differences have been identified between HER2-low and HER2-null tumors after adjusting for HR expression. Most HER2-low/HR+ tumors are intrinsically classified as either luminal A or B (90%), while the majority of HER2-low/TNBCs are categorized as basal-like (85%) (12, 13, 21, 22, 26–29). In a comprehensive study exploring PAM50 gene expression, Schettini et al. observed consistent distributions of intrinsic subtypes between IHC1+ and IHC2+/ISH- subgroups: Luminal A was the most prevalent (49% vs. 54%), followed by Luminal B (28% vs. 30%) and Basal-like (15% vs. 10%) (12). Interestingly, Agostinetto et al. noted that the intrinsic subtype distribution in HER2-low/HR+ BC more closely resembles HER2-null/HR+ than HER2-low/TNBC, suggesting that the biological differences are primarily driven by HR expression rather than HER2-low status (21).

In our cohort, when evaluating the clinicopathologic surrogates of intrinsic subtypes, 92% of cases were classified as luminal-like BC (mostly B-like) and 8% as TNBC. These results are consistent with previous reports indicating that 10-13% of HER2-low BC are histologically categorized as TNBC, while 87-90% are HR+ (24, 25, 30). Overall, the HER2-low category predominantly presents in HR+ tumors, constituting at least two-thirds, in contrast to TNBC, where it comprises approximately one-third (12, 24).

The predominance of early−stage disease in this cohort likely reflects system−level factors, including the organized mammography screening program in Portugal and expedited diagnostic pathways typical of a private academic tertiary setting, which together favor earlier detection and referral compared to general community settings.

The standard of care for early-stage, high-risk HER2-negative BC usually includes (neo)adjuvant chemotherapy (31). While traditional anti-HER2 drugs like trastuzumab and pertuzumab significantly improve outcomes for HER2-positive BC (32), their efficacy does not extend to HER2-negative disease, including the HER2-low population (33, 34). Integrating these therapies into standard chemotherapy in early-stage (33) or advanced (34) HER2-low BC has not shown survival benefits.

In our NACT-treated cohort, about half were IHC1+ and half IHC2/ISH-. The demographic and clinicopathological features were balanced between the two subgroups, except for the median age and proportion of postmenopausal women, which were lower in the IHC1+ subgroup. As expected, patients selected for this strategy were mostly young individuals with higher stage BC and a more aggressive phenotype—mainly luminal B-like (58%) and TNBC (33%)— exhibiting higher baseline Ki67 index levels and/or higher histologic grade. Regimen distribution was largely balanced between HER2−low subgroups, with the majority (86%) receiving AC-taxane based chemotherapy and only six patients on alternative regimens.

The high proportion of HR+ BC may account for the overall low pCR rate of 21%, aligning with previous studies that underline this as an unmet need. In fact, existing data suggest that only around 1 in 5 patients with early-stage BC achieve pCR after NACT, with the highest pCR rates observed among HER2-positive and TNBC cases, while HR+/HER2-negative tumors usually show the lowest (35, 36). It is also recognized that patients with HER2-positive and TNBC who achieve pCR have significantly better long-term outcomes compared with those who do not. For HR+/HER2-negative BC, where pCR is less frequent and adjuvant endocrine therapy is the cornerstone of systemic treatment, there appears to be a trend towards improved survival, especially in higher-grade and/or luminal B subtypes (36). Moreover, studies on HER2-negative BC suggest that HER2-low tumors generally achieve lower pCR rates compared to HER2-null tumors after NACT, although these differences do not always reach statistical significance in multivariable logistic regression analyses (2, 13, 22, 25, 30, 37, 38).

One possible explanation for this is that even low-range HER2 expression, as opposed to none, may contribute to resistance to NACT due to crosstalk between HER2 and other oncogenic pathways, including ER signaling (30, 33). Notably, HER2-low tumors generally exhibit a higher HER2 copy number compared to HER2-null tumors, particularly in HR-positive disease. The highest levels are often found in IHC 2+ tumors, followed by IHC 1+ and IHC 0 (12, 21).

In this cohort, despite overall similar patient and tumors characteristics across subgroups, IHC2+/ISH- status was significantly associated with lower odds of achieving pCR compared to IHC1+ among patients treated with NACT. Conversely, while higher baseline Ki-67 levels and ER-negative status were associated with higher pCR rates in the univariable logistic regression analysis, these associations did not remain statistically significant after adjustment in the pre−specified multivariable model. Given the limited number of pCR events, the adjusted multivariable analysis is interpreted as a supportive exploration of potential confounding rather than as the primary inferential basis for the secondary endpoint. While the low events−per−variable ratio warrants caution, the alignment between univariable and adjusted estimates supports the observed HER2 subgroup effect.

It is important to consider that age imbalance might have affected our results, as the IHC1+ subgroup included a younger population. However, large retrospective studies indicate that pCR rates are generally comparable across age groups in early-stage BC treated with NACT. Conversely, younger women with TNBC appear to achieve pCR more frequently, potentially due to a higher prevalence of BRCA mutations and tumour-infiltrating lymphocytes (TIL)-rich tumors with enhanced chemosensitivity, and axillary downstaging rates are typically higher among younger women compared to their older counterparts (50). In our cohort, since the majority (67%) of NACT-treated patients were not TNBC, the potential age-related bias influencing our findings may be attenuated. Furthermore, in logistic regression analysis, lower age was not significantly associated with increased odds of achieving a pCR. However, given the retrospective cohort design of our study, we cannot exclude a trend toward more effective NACT protocols and higher dose-intensity in younger patients, potentially contributing to higher pCR rates in the IHC1+ subgroup.

In a pre−specified exploratory analysis, no differences were detected between HER2−low subgroups in nodal−status changes after NACT. Although a numerically higher conversion from cN+ to ypN0 was observed in IHC 1+ versus IHC 2+/ISH−, this difference was not statistically significant and should be viewed as hypothesis−generating given limited power. Potential subgroup differences in axillary downstaging could have implications for surgical de−escalation and prognosis and warrants evaluation in larger cohorts.

Looking ahead, the future appears promising as we explore innovative treatment strategies. Recent advances in HER2-targeting antibody-drug conjugates (ADCs), such as trastuzumab deruxtecan (T-DXd), have revolutionized the treatment landscape for metastatic BC (mBC) (39). Equipped with potent cytotoxic payloads, high drug-to-antibody ratios, and the ability to elicit bystander effect, these novel ADCs exhibit significant antitumoral activity in HER2-low tumors without gene amplification (15, 29, 39, 40). Within the HER2-negative BC spectrum, even low HER2 expression may now provide independent prognostic insights, challenging the traditional view that only tumors with HER2-positive BC qualify for HER2-directed therapies. This emphasizes the importance of understanding the clinicopathological characteristics of this group, particularly the predictive implications of varying HER2 IHC levels (41, 42).

The activity of T-DXd led to its regulatory approval and integration into current treatment guidelines of HER2-low mBC (2), paralleled by promising preliminary results from novel HER2-targeting ADCs such as trastuzumab duocarmazine (43), disitamab vedotin (44), MRG002 (45), and SHR-A1811 (46), which may follow a similar path. Whether this new class of drugs will achieve similar benefits in early-stage HER2-low BC remains to be seen, although initial studies with T-DXd in this setting have yielded encouraging results (47).

Future studies may also benefit from incorporating serum biomarkers and biochemical parameters (e.g., lactate dehydrogenase) (52), alongside emerging cellular signaling markers, which could enhance risk stratification and provide deeper biological insights into HER2−low tumor behavior and treatment response.

## Limitations

Our study has several limitations. First, as a single-center retrospective analysis, it is vulnerable to a limited sample size and potential bias, particularly given the high prevalence of early-stage BC cases in our institution. Second, despite strict adherence to ASCO/CAP guidelines (3) and the involvement of an experienced team of pathologists, the absence of central pathological review precludes conclusions about interobserver discordance in HER2-low status. Additionally, the study focused on pre−specified tissue−based clinicopathological variables and did not collect serum biomarkers or other biochemical indicators that might provide additional prognostic or biological information relevant to HER2−low breast cancer. We endeavored to minimize bias by including over 200 patients to increase the reliability of our results. Nonetheless, particularly in patients undergoing NACT, a larger cohort would allow for more robust multivariable logistic regression analysis and narrower confidence intervals, enhancing the reliability of the findings. The low events-per-variable ratio introduces a risk of overfitting. Accordingly, the adjusted analysis was exploratory and supportive of the univariable findings, which remain the main descriptive evidence for the secondary endpoint. The small number of patients using other regimens than dose−dense doxorubicin-cyclophosphamide sequence followed by paclitaxel, precluded the ability to model regimen effects in multivariable analysis without overfitting. The exploratory nodal−status analysis is descriptive, and results should be interpreted cautiously. No additional small−sample sensitivity procedures were added in order to preserve the pre−specified analytic scope and avoid overextending inference beyond the available data. Lastly, since patients were collected after 2018, the follow-up period was relatively short, and pCR was used as a surrogate endpoint. Longer observation will be necessary to draw definitive conclusions about survival outcomes. On the other hand, the study did not include patients after 2020, thereby excluding the period when anti-PD-1/PD-L1 inhibitors became part of the standard care for TNBC, which could have resulted in higher pCR rates (48).

## Conclusion

In conclusion, our study did not document significant clinicopathological differences between the IHC1+ and IHC2+/ISH- subgroups, similar to previous published data that failed to establish HER2-low as a unique entity among HER-negative BC. However, there was a significant association between HER2-low IHC2+ status and lower odds of achieving pCR after NACT in both univariable and multivariable analysis. This indicates that HER2-low status may independently influence the likelihood of pCR, despite similarities in other characteristics. While both higher baseline Ki67 levels and ER-negative status (vs. ER-positive) showed a trend towards higher pCR rates, these associations were not significant in the multivariable analysis.

Although HER2-low IHC 1+ and 2+/ISH- BC appear to fall within the same spectrum, there seems to be a gradual increase in low-level HER2 expression that may influence treatment response. In addition, previous data indicate that for patients who achieve pCR, the prognosis appears favorable irrespective of HER2-null or HER2-low status (35). While this alone should not influence neoadjuvant treatment decisions with current chemotherapy regimens, HER2-low status could be integrated into future multivariable prediction models for pCR in HER2-negative breast cancer, potentially informing stratified treatment approaches.

The HER2-low category encompasses a heterogeneous group of tumors whose profiles seem to be strongly influenced by HR expression. Therefore, other predictors beyond HER2 IHC status should be considered. Nonetheless, the emergence of post-neoadjuvant strategies based on pCR outcomes, as already established in both HER2-postive and negative BC (49), suggests that these findings could have broader future implications.

Looking ahead, HER2-low BC is a challenging population in whom to test new HER2-targeting ADCs, where outcomes can be driven by the presence of low-level HER2 expression. Given the efficacy of ADCs in HER2-low mBC after multiple prior lines of treatment, and the promising results from initial neoadjuvant trials in early-stage BC, several questions arise. A phase III randomized trial is still needed to compare novel anti-HER2 ADCs with standard NACT in early-stage BC. Additionally, efforts should focus on understanding whether varying levels of HER2 expression can predict treatment response to HER2-directed ADCs. Finally, integrating more sensitive and reproducible HER2 assays into clinical practice may, not only support future research, but also refine the accuracy of routine HER2 classification and improve patient selection for targeted therapies.

## Data Availability

The data analyzed in this study is subject to the following licenses/restrictions: Pseudo-anonimization, dataset from EMR, only shared among investigators. Requests to access these datasets should be directed to jorgealvescorreia.mail@gmail.com.
